# Characterization of the Binding and Inhibition Mechanisms of a Novel Neutralizing Monoclonal Antibody Targeting the Stem Helix Region in the S2 Subunit of the Spike Protein of SARS-CoV-2

**DOI:** 10.3390/vaccines13070688

**Published:** 2025-06-26

**Authors:** Selene Si Ern Tan, Ee Hong Tam, Kah Man Lai, Yanjun Wu, Tianshu Xiao, Yee-Joo Tan

**Affiliations:** 1 Infectious Diseases Translational Research Programme, Department of Microbiology and Immunology, Yong Loo Lin School of Medicine, National University of Singapore, Singapore 117545, Singapore; s_tan@nus.edu.sg (S.S.E.T.);; 2 School of Biological Sciences, Nanyang Technological University, Singapore 637551, Singapore; eehong.tam@ntu.edu.sg (E.H.T.); yanjun001@e.ntu.edu.sg (Y.W.); tianshu.xiao@ntu.edu.sg (T.X.)

**Keywords:** SARS-CoV-2, spike protein, monoclonal antibodies

## Abstract

Background/Objectives: For viral entry into host cells, the spike (S) protein of coronavirus (CoV) uses its S1 domain to bind to the host receptor and S2 domain to mediate the fusion between virion and cellular membranes. The S1 domain acquired multiple mutations as the severe acute respiratory syndrome coronavirus 2 (SARS-CoV-2) evolved to give rise to Variant of Concerns (VOCs) but the S2 domain has limited changes. In particular, the stem helix in S2 did not change significantly and it is fairly well-conserved across multiple beta-CoVs. In this study, we generated a murine mAb 7B2 binding to the stem helix of SARS-CoV-2. Methods: MAb 7B2 was isolated from immunized mouse and its neutralization activity was evaluated using microneutralization, plaque reduction and cell–cell fusion assays. Bio-layer interferometry was used to measure binding affinity and AlphaFold3 was used to model the antibody–antigen interface. Results: MAb 7B2 has lower virus neutralizing and membrane block activities when compared to a previously reported stem helix-binding human mAb S2P6. Alanine scanning and AlphaFold3 modeling reveals that residues K1149 and D1153 in S form a network of polar interactions with the heavy chain of 7B2. Conversely, S2P6 binding to S is not affected by alanine substitution at K1149 and D1153 as indicated by the high ipTM scores in the predicted S2P6-stem helix structure. Conclusions: Our detailed characterization of the mechanism of inhibition of 7B2 reveals its distinctive binding model from S2P6 and yields insights on multiple neutralizing and highly conserved epitopes in the S2 domain which could be key components for pan-CoV vaccine development.

## 1. Introduction

Severe acute respiratory syndrome coronavirus 2 (SARS-CoV-2) is a novel coronavirus that emerged in late 2019 and caused a devastating coronavirus disease 2019 (COVID-19) pandemic. Consistent with SARS-CoV and Middle Eat respiratory syndrome coronavirus (MERS-CoV), SARS-CoV-2 belongs to the genus betacoronavirus (Beta-CoV) that comprise a positive-sense, single-stranded enveloped RNA and four structural proteins, namely the spike (S) protein, membrane (M) protein, envelope (E) protein and nucleocapsid (N) protein [1]. Over the past four years, SARS-CoV-2 has acquired multiple genetic mutations and rapidly evolved from the wildt-ype strain isolated in Wuhan, China, to several variants with superior properties such as greater transmissibility and immune evasion [2]. The highly contagious omicron variant was first discovered in late November 2021, with at least 60 mutations compared to the original Wuhan isolate. Succeeding subvariants of Omicron including BA.5, XBB.1, EG.5.1 and JN.1 with increased viral fitness due to the mutations on S protein have gained dominance globally and caused severe complication and mortality in vulnerable populations [3,4].

The S protein plays a pivotal role in facilitating the entry of SARS-CoV-2 into the host cells. It is a class I fusion transmembrane glycoprotein that forms a clove-shaped homotrimer protruding from the viral surface. The S protein has a total length of 1273 amino acids and comprises the S1 and S2 subunits. The S1 subunit consists of an N-terminal domain (NTD) and a receptor-binding domain (RBD), whereas the S2 subunit is composed of a fusion peptide (FP), heptapeptide repeat 1 (HR1), HR2, transmembrane domain and cytoplasmic domain [5]. The S protein mediates viral entry by recognizing and binding to the host angiotensin-converting enzyme 2 (ACE2) receptor via RBD. Binding to the ACE2 receptor leads to conformational changes in the S1 subunit and exposes the S2’cleavege site, which is subsequently cleaved by host proteases. Following cleavage, the S1 subunit is dissociated and fusion between virus and host cell membrane is induced to allow the release of viral genome into the host cell [6].

A range of antiviral agents like small molecular inhibitors, monoclonal antibodies (mAbs) and vaccines that target S protein, especially the RBD-ACE2 interaction, have been developed. However, Omicron and its subvariants harbor multiple mutations in RBD that result in immune evasion from preexisting immunity induced by previous natural infection, vaccines and even therapeutic mAbs which are designed to block viral entry mediated by the S protein [7]. Besides its usefulness for therapy, neutralizing anti-S mAbs can also be used as pre-exposure prophylaxis for some immunocompromised individuals who do not respond adequately to approved COVID-19 vaccines and are at higher risk of prolonged illness and poor recovery outcomes [8]. Similarly, pre-exposure prophylaxis may be an alternative for the low number of people who developed severe side-effects to vaccination [9]. For example, several systematic reviews and meta-analyses have shown that pre-exposure prophylaxis of tixagevimab + cilgavimab, which is a combination of two anti-S neutralizing mAbs, has been clinically effective in reducing infection risk and severity of illness in immunocompromised patients [10,11].

Thus far, all the approved therapeutic mAbs for COVID-19 are targeting the S1 subunit but research has shown that neutralizing mAbs targeting S2 are more likely to retain neutralizing potency against new SARS-CoV-2 variants [12,13]. This is because the S2 subunit has remained fairly well-conserved among the variants and represents an ideal target for the design and development of cross-protective antiviral mAbs. In our previous study, we reported an immunogenic domain in the S2 subunit of SARS-CoV, comprising HR2 as well as the linker between HR1 and HR2. This domain is highly conserved in SARS-CoV-2 and a mAb; 1A9 raised against this immunogenic fragment is able to cross-react with the SARS-CoV-2 S protein [14,15,16]. In the present study, we aimed to generate neutralizing mAbs that target the S2 subunit of the S protein using the corresponding fragment in SARS-CoV-2 and identify the neutralizing epitopes for the development of vaccine and therapeutic agents against the emerging variants.

## 2. Materials and Methods

### 2.1. Cells and Virus

The Vero-E6 cell was purchased from American Type Culture Collection (ATCC; Manassas, VA, USA) and cultured in Dulbecco’s Modified Eagle’s Medium (DMEM; Hyclone, Logan, UT, USA) supplemented with 10% fetal bovine serum (FBS; Hyclone) and 1% non-essential amino acids (NEAA; Thermo Fisher Scientific, Waltham, MA, USA). The 293FT cell was purchased from Invitrogen (Carlsbad, CA, USA) and cultured in DMEM supplemented with 10% FBS, 1% NEAA, 100 units/mL penicillin–streptomycin and 500 µg/mL geneticin (Thermo Fisher Scientific). Vero-E6 and 293FT cells were maintained at 37 °C with 5% CO_2_. The FreeStyle 293-F cell was purchased from Thermo Fisher Scientific and cultured in F17 expression media supplemented with 2% L-glutamine and 1% Pluronic F68 (Thermo Fisher Scientic). The 293-F cell was grown in incubator shaker at 37 °C, 5% CO_2_ and 95 rpm. SARS-CoV-2 (Omicron subvariant EG.5.1.1, hCoV-19/Singapore/NUS0001/2023, GISAID accession: EPI_ISL_19016298) was isolated in a previous study [17].

### 2.2. Construction of Plasmids for Expression in Mammalian Cells

Beta-CoVs S protein fragment genes were codon-optimized and generated by gene synthesis (Bio Basic Asia Pacific, Singapore) according to GenBank accession number: SARS-CoV-2 WT (YP_009724390.1), OC43 (L14643.1), HKU5 (AGP04943.1), MERS-CoV (KT357812.1). To obtain the S fragment gene of mouse hepatitis virus (MHV), RNA was extracted from MHV (VR-764, ATCC) followed by reverse transcription to synthesize cDNA. The desired MHV S fragment gene was amplified using specific primers via PCR. The S fragment genes of beta-CoVs were then cloned into the pXJ40-Myc expression vector for expression of the Myc-tagged S-protein fragment. To generate alanine mutants, two-round PCR site-directed mutagenesis was performed on the wild-type SARS-CoV-2 S fragment using specific primers and Q5^®^ High-Fidelity DNA Polymerase (New England Biolabs, MA, USA). The PCR products were then cloned into the pXJ40-Myc expression vector using BamHI and XhoI restriction sites.

### 2.3. Generation and Purification of Monoclonal Antibodies

BALB/c mice were immunized with the recombinant SARS-CoV-2 WT S protein fragment (residues 1048 to 1206), expressed and purified from the *E. coli* expression system using the method previously described for SARS-CoV-1 [14]. Animal experiments were performed according to the approved institutional animal care and use committee (IACUC) protocol (R22-0062, approved on 29 July 2022) at the National University of Singapore. Hybridomas were obtained by fusion of SP2/0 myeloma cells and splenocytes from the immunized mice by using the ClonalCell-HY Complete kit (StemCell Technologies, BC, Canada) according to the manufacturer’s protocol. Hybridomas were screened for secretion of S-specific mAbs by enzyme-linked immunosorbent assay (ELISA) using a recombinant whole ectodomain of SARS-CoV-2 S protein (cat#40589-V08B1, Sino Biological, Beijing, China). The variable light and heavy domains of mAbs produced by selected hybridomas were sequenced and plasmids that expressed recombinant mAbs with human IgG1 constant region were constructed (Biointron, Shanghai, China). The published sequences [18] of S2P6 mAb were constructed in a similar way. The recombinant mAbs were expressed in a 293-F expression system and purified using affinity chromatography, as described previously [19].

### 2.4. Transient Transfection and Western Blot Analysis

293FT cells seeded on 60 mm dishes were transiently transfected with S fragment of indicated beta-CoVs or S fragment of SARS-CoV-2 WT with alanine substitutions using X-tremeGENE HP DNA transfection reagent (Roche, Basel, Switzerland) according to the manufacturer’s protocol. At 24 h post transfection, cells were washed twice with PBS and harvested using cell scrapers in RIPA buffer (50 mM Tris-HCl (pH 8.0), 150 mM NaCl, 0.5% NP-40, 0.5% sodium deoxycholate and 1 mM phenylmethylsulfonyl fluoride (PMSF)). Cell lysates were incubated on ice for 30 min with vortexing occasionally. Cell lysates were centrifuged at 13,000 rpm at 4 °C to remove cell debris and concentration of cell lysates were quantified using Pierce™ Detergent Compatible Bradford Assay Kit (Thermo Fisher Scientific) according to the manufacturer’s protocol. For Western blot analysis, 10 µg of cell lysates were boiled in 1× Laemmli SDS sample buffer. Proteins were separated by SDS-PAGE and transferred to nitrocellulose membrane (Bio-Rad, Hercules, CA, USA). The membrane was blocked in 5% skimmed milk in Tris-buffered saline with 0.05% Tween 20 (TBST) for 1 h at room temperature (RT) and incubated with mAbs overnight at 4 °C. After three washes with TBST, the membrane was incubated with horseradish peroxidase (HRP)-conjugated secondary antibody (Thermo Fisher Scientific) for 1 h at RT. After three washes with TBST, bound antibodies were visualized with enhanced chemiluminescence (ECL) substrate (Thermo Fisher Scientific) using the ChemiDoc MP Imaging System (Bio-Rad).

### 2.5. Immunofluorescence Analysis (IFA)

Vero-E6 cells seeded on a coverslip were mock-infected or infected with SARS-CoV-2 at a multiplicity of infection (MOI) of 5 in DMEM supplemented with 2% FBS. At 24 h post infection (hpi), infected cells were fixed with 4% paraformaldehyde (PFA) and permeabilized with 0.2% Triton X-100 in PBS. Cells were incubated with IFA buffer (10% FBS in PBS) for 30 min followed by staining with indicated mAbs at 2 µg/mL in IFA buffer for 1 h. After being washed with PBS three times, cells were incubated with Alexa-Fluor (AF) 488-conjugated goat anti-human IgG antibody and 4′,6-diamidino-2-phenylindole (DAPI) (Thermo Fisher Scientific) in IFA buffer for 1 h. After being washed with PBS three times, the coverslips were mounted on a microscope slide with ProLong™ Gold Antifade Mountant (Thermo Fisher Scientific) and images were captured using a fluorescent microscope.

### 2.6. ELISA and Peptide Mapping

The whole ectodomain of the SARS-CoV-2 WT S protein with His tag ((#40589-V08B1, Sino Biological) was diluted with coating buffer (0.1 M NaHCO_3_, 34 mM Na_2_CO_3_) and 50 ng of protein was coated into individual wells of a 96-well plate (Thermo Fisher Scientific) overnight at 4 °C. Plates were washed three times with 0.05% Tween 20 in PBS (PBST) and blocked with blocking buffer (5% FBS in PBST) for 1 h incubation at 37 °C. The mAbs were diluted with blocking to indicated concentration and added to desired wells for 2 h at 37 °C. HRP-conjugated secondary antibody diluted in blocking buffer was added for 1 h incubation to each well after three washes with PBST. Visualization of bound antibodies was performed by the addition of 3,3′,5,5′-tetramethylbenzidine (TMB) substrate (Thermo Fisher Scientific) for 2 min and the reaction was stopped with 2 N sulphuric acid. Optical density at 450 nm (OD450 nm) was measured using Tecan Infinite M1000 plate reader (Männedorf, Switzerland). For peptide mapping, 10 µg/mL of synthetic 20-mer overlapping peptides (WuXi AppTec (Hong Kong) Limited) were coated on 96 well plates using coating buffer overnight at 4 °C. Coated plates were washed three times with PBST and blocked with blocking buffer for 1 h at 37 °C. A measure of 30 ng/mL of mAbs diluted in blocking buffer was added to the desired wells for 2 h incubation at 37 °C. After three washes with PBST, HRP-conjugated secondary antibody diluted in blocking buffer was added, followed by 1 h incubation at 37 °C. The plates were washed again with PBST and bound antibodies were visualized after the addition of TMB and 2 N sulphuric acid. Absorbance at 450 nm was determined using Tecan Infinite M1000 plate reader.

### 2.7. Microneutralization Test (MNT)

The Vero-E6 cell was seeded in 96-well plate and incubated for 24 h. A measure of 15,000 pfu/mL of SARS-CoV-2 (Omicron subvariant EG.5.1.1) was mixed with mAbs at indicated concentrations in a 1:1 ratio and incubated for 1 h at RT. The mAb/virus mixtures were added to the cells and incubated for 1 h at 37 °C. After 1 h incubation, the inoculum was removed. The same concentrations of mAbs diluted using 2% FBS DMEM were added to the infected cells. At 24 hpi, infected cells were fixed with 4% PFA and permeabilized with 0.2% Triton X-100. The cells were incubated with blocking buffer (10% FBS in PBS) for 1 h at RT and stained with 0.1 µg/mL of a home-made anti-SARS-CoV-2 N mAb (clone 7A7, Liew et al., 2025 [20]) for 2 h at RT followed by three washes with PBS. Cells were incubated with HRP-conjugated secondary antibody for 1 h followed by three washes with PBS. The bound antibody was visualized after the addition of TMB and 2 N sulphuric acid. Absorbance at 450 nm was determined using a Tecan Infinite M1000 plate reader. The percentage (%) of MNT was calculated according to a previous study [21]. The % MNT is equal to 100 − [(absorbance of sample wells − average of mock-infected wells)/(absorbance of PBS control wells − average of mock-infected wells) × 100].

### 2.8. Plaque Reduction Neutralization Test (PRNT)

The Vero-E6 cell was seeded in 12-well plates and incubated for 24 h. A measure of 240 pfu/mL of SARS-CoV-2 (Omicron subvariant EG.5.1.1) was mixed with mAbs at indicated concentrations in a 1:1 ratio and incubated for 1 h at RT. The mAb/virus mixtures were added to the cells and incubated for 1 h at 37 °C, with rocking at 15 min intervals. The inoculum was removed and an overlay (1.2% Avicel (FMC BioPolymer, Philadelphia, PA, USA) and MEM supplemented with 1% FBS) was added into each well. At 3 days post-infection (dpi), the overlay was removed and cells were fixed with 10% formalin for 1 h followed by staining with 0.1% crystal violet. The percentage of plaque reduction was calculated according to a previous study [22]. The % PRNT is equal to 100 × [1 − (average number of plaques of sample wells/average number of plaques of PBS control wells)].

### 2.9. Cell–Cell Fusion Assay

Membrane fusion mediated by the spike protein was quantified by a beta-galactosidase based cell–cell fusion assay. HEK293T cells were transiently transfected using Polyethylenimine (PEI) (Polysciences, Warrington, PA, USA) to express SARS-CoV-2 B.1 S protein or ACE2. The B.1 lineage, which was dominant globally in early 2020, was chosen because it contains the G614 mutation in S and this mutation enhances membrane fusion. Briefly, plasmid encoding the Spike (0.05 µg) or ACE2 (1.5 µg) cloned in an expression vector with CMV promotor was mixed with plasmid of alpha or omega fragment (1.5 µg) of beta-galactosidase, diluted in DMEM and incubated with PEI at a PEI-to-DNA ratio of 4:1. The DNA-PEI mixture was added to HEK293T cells with 70–80% confluence cultured in 6-well plate, followed with 6 h incubation. At the end of incubation, culture medium was removed and replaced with fresh complete DMEM, to allow protein expression for 24 h. The Spike/alpha fragment or ACE2/omega fragment expressing cells were harvested by centrifugation and resuspended in fresh complete DMEM with cell density adjusted to 2.5 × 10^5^/mL. Antibody, 7B2 or S2P6, or PBS buffer was added to 50 µL S/alpha fragment cells, seeded into a 96-well plate for a 1 h incubation under 37 °C and 5.0% CO_2_. Subsequently, 50 µL of ACE2/omega fragment cells were added to the mixture, followed with a 3 h incubation under 37 °C and 5.0% CO_2_ to allow cell–cell fusion to develop. To quantify membrane fusion activity, 100 µL of substrate (Gal-screen, Thermo Fisher) was added to the 96-well plate and incubated for 90 min at room temperature to allow luminescence development. Luminescence was measured using a BioTek Synergy H1 microplate reader.

### 2.10. Bio-Layer Interferometry (BLI) to Quantify Binding Kinetics

Binding kinetics was quantified with BLI assay. A synthetic peptide of stem helix (1141–1161), which is designed based on SARS-CoV-2 WT S, with biotin at C terminus was immobilized on SA sensor (Gator Bio, Palo Alto, CA, USA). Immobilization was carried out following the standard protocol recommended by the manufacturer, with the stem helix peptide in PBS at 10 μg/mL. Antibody, S2P6 or 7B2, was diluted in running buffer (100 mM HEPES, 500 mM NaCl, 0.005% Tween20 and 0.1% BSA) at concentrations varied between 1 and 100 nM. The sensors immobilized with stem helix were dipped in the antibody to allow the detection of antibody association, followed by dipping the sensors into running buffer to detect the dissociation step. Data analysis was performed using 1:1 global fitting model with GatorPrime analysis software (version 2.15.5.1221).

### 2.11. Structure Prediction by AlphaFold3

Amino acid sequences of 7B2 or S2P6 single-chain variable fragment (scFv) and the stem helix (1145–1158) were applied for AlphaFold3 [23] prediction. Briefly, the scFv consisted of heavy chain variable region and light chain variable region of antibody spaced with a 20 amino acid GS linker. The sequences of the scFv and stem helix (with and without N-acetylglucosamine at N1158) were uploaded as two entities to AlphaFold3 for structure prediction of the complex, with confidence scores generated to indicate prediction quality.

### 2.12. Data Analysis

The 50% of effective concentration (EC50) and 50% of inhibitory concentration (IC50) were calculated by non-linear, dose-dependent regression analysis using GraphPad Prism 10 software. Multiple group comparison was analyzed by two-way analysis of variance (ANOVA) followed by Dunnett’s multiple comparison test, using * *p* < 0.05, ** *p* < 0.01, *** *p* < 0.001 and **** *p* < 0.0001 to indicate the statistical significance, with * *p* < 0.05 being considered statistically significant.

## 3. Results

### 3.1. Generation of mAb 7B2 from Mice Immunized with S2 Subunit Fragment of SARS-CoV-2 S Protein

**Figure 1:**
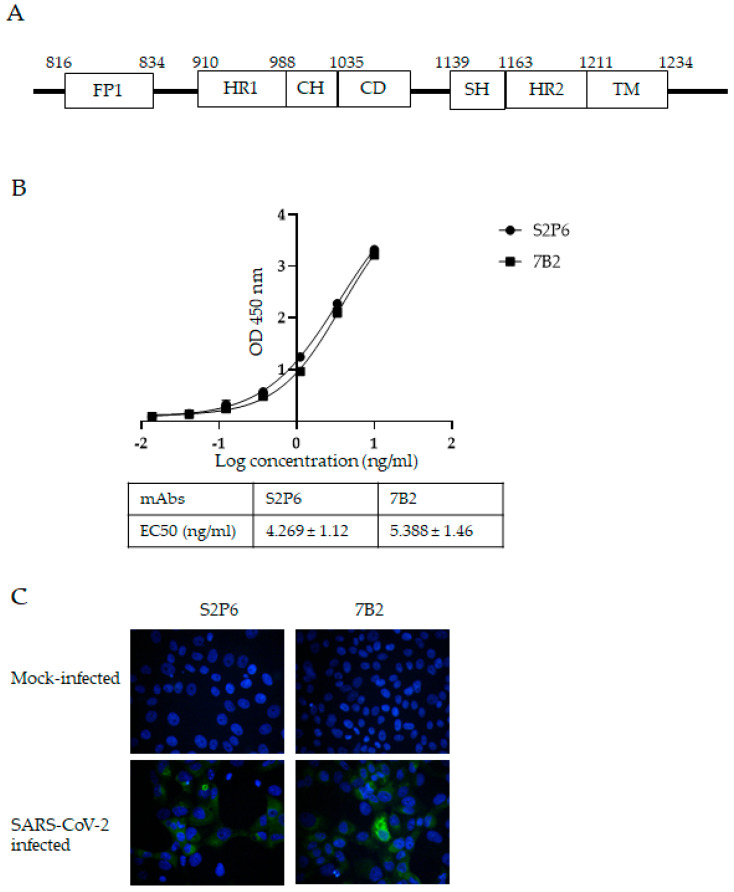
Generation of S protein-specific mAbs with S2 subunit fragment of SARS-CoV-2 S protein. (**A**) Schematic diagram showing functional motifs in S2 subunit of SARS-CoV-2 S protein. Abbreviations: FP-fusion peptide; HR1-heptad repeat 1; CH-central helix; CD-connector domain; SH-stem helix; HR2-heptad repeat 2; TM-transmembrane. (**B**) Binding of mAbs S2P6 and 7B2 to SARS-CoV-2 S protein and their respective EC50 were determined by ELISA. A representative plot from three independent experiments is shown and error bars correspond to standard deviations (SD) of each mAb experiment carried out in triplicate. (**C**) Vero-E6 cells were mock-infected or infected with SARS-CoV-2 at MOI of 5. At 24 hpi, infected cells were stained with mAbs S2P6 and 7B2 at 2 µg/mL, followed by AF-488-conjugated secondary antibody. Nuclei were counterstained with DAPI. Images were captured using fluorescent microscope.

As shown in Figure 1A, the S2 domain of SARS-CoV-2 contains different functional motifs [24]. Our previous work revealed an immunogenic region in the S2 subunit of SARS-CoV S protein covering the entire HR2 domain and its upstream region. Polyclonal and monoclonal antibodies raised against this region neutralized in vitro infection with SARS-CoV [14,15,16]. In the present study, the corresponding residues 1048 to 1206 (which contains the HR2 and stem helix (SH) upstream of HR2) of SARS-CoV-2 WT S protein were expressed in an *E. coli* expression system and used to immunize mice. The residue numbering is based on SARS-CoV-2 wild-type (WT). Sequence alignment shows that this region is well-conserved among SARS-CoV-2 WT and VOCs, with all the VOCs showing 98 to 100% identity to WT (Supplementary Table S1). In comparison, residues 686 to 1047, which contain the fusion peptide and HR1, show more evolutionary change, with some VOCs having ~96% identity with WT. An anti-S mAb named 7B2 was isolated from the immunized mouse and chimeric recombinant 7B2 mAb with human IgG constant region and was produced and purified for subsequent characterization. The binding of 7B2 to the recombinant S protein was determined by ELISA. S2P6, a human mAb isolated from a COVID-19 patient with broad neutralization breadth against beta-CoVs was used as a positive control [18]. S2P6 and 7B2 bound the recombinant S protein with EC50 at 4.269 ng/mL and 5.388 ng/mL, respectively (Figure 1B). In early 2023, XBB subvariants replaced the circulating Omicron subvariants and gained dominance globally. It rapidly evolved into multiple subvariants and sub-lineages. EG.5 is a descendent lineage of XBB.1.9.2, which carries an additional F456L mutation in the S protein. Due to its high global prevalence, EG5 and its descendent lineages were designated as a VOI by World Health Organization (WHO) on 8 August 2023 [25]. In the present study, we demonstrated that S2P6 and 7B2 at concentration of 2 µg/mL were able to detect the S protein expressed in Vero-E6 cells infected by the Omicron subvariant EG.5.1.1 (Figure 1C).

### 3.2. Neutralization Activity of mAbs Against Omicron Subvariant EG.5.1.1

**Figure 2:**
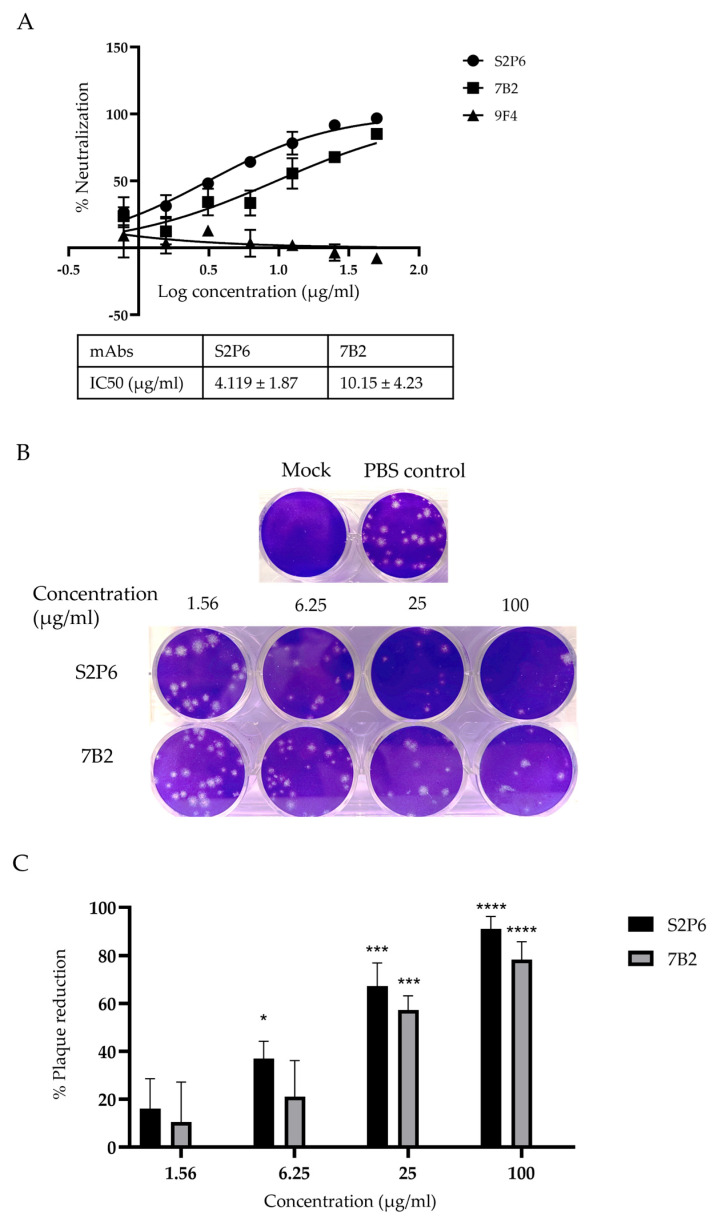
Neutralization activity of anti-S mAbs was determined by MNT and PRNT. (**A**) Percentage of neutralization by mAbs at indicated concentrations and their respective IC50 were determined by MNT. A representative plot from three independent experiments is shown and error bars correspond to standard deviations of each mAb experiment carried out in triplicates. (**B**,**C**) Percentage of plaque reduction in mAbs at indicated concentrations was determined by PRNT. Bar graph represents the mean percentage of plaque reduction across three independent experiments and error bars correspond to SD across the three experiments. For each mAb, the plaque reduction at stated concentration is compared to the PBS control group for statistical analysis. * *p* < 0.05, *** *p* < 0.001, **** *p* < 0.0001, compared with PBS control group.

The neutralization activity of mAbs was assessed via MNT and the results show that S2P6 and 7B2 neutralized the infection of EG.5.1.1 in Vero-E6 cells with IC50 at 4.119 µg/mL and 10.15 µg/mL, respectively, (Figure 2A). No neutralization was observed for an irrelevant mAb 9F4, which binds to influenza A hemagglutinin [26]. Based on MNT, S2P6 showed more potent viral neutralization compared to 7B2. This finding is consistent with PRNT, which showed that S2P6 exhibited a higher percentage of plaque reduction compared to 7B2 at concentrations of 6.25 µg/mL, 25 µg/mL and 100 µg/mL (Figure 2B,C).

### 3.3. Mapping the Epitope of mAb 7B2

**Figure 3:**
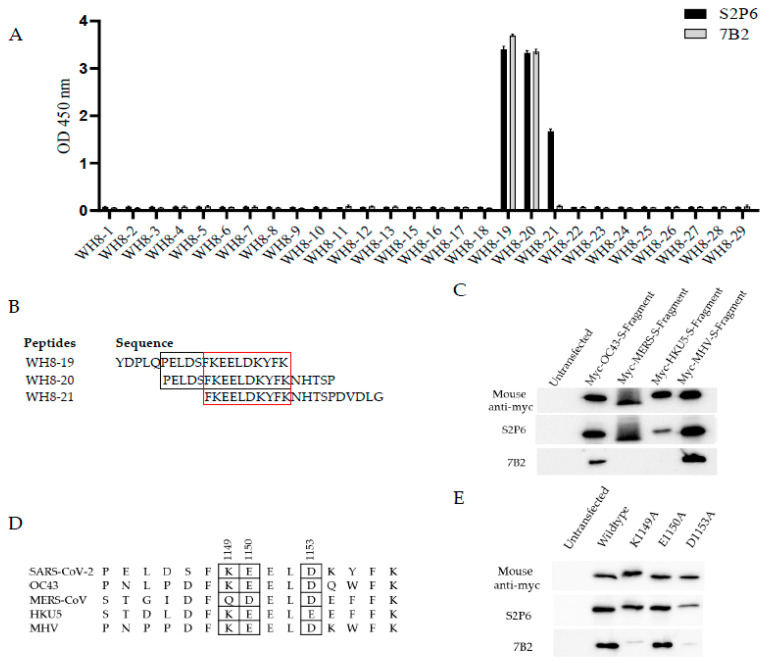
Epitope mapping for 7B2. (**A**) Here, 20-mer peptides with 15 overlapping amino acids from extended HR2 region of SARS-CoV-2 S protein were used to map the epitopes. Binding of mAbs to peptide was determined by ELISA. A representative plot from two independent experiments is shown. Data represents mean readings, with error bars showing SD of triplicate wells. (**B**) Sequence of peptides WH8-19, WH8-20 and WH8-21 were aligned and the minimal binding sequence for S2P6 was denoted in red box while for 7B2 was denoted in black box. (**C**) Cross-reactivity of mAbs to S fragment of beta-CoVs were determined by Western blot analysis. 293FT cells were transfected with myc-tagged S fragments of beta-CoV including HCoV-OC43, MERS-CoV, BtCoV HKU5 and MHV. After 24 h, cell lysates were harvested and subjected to Western blot analysis. Anti-myc antibody was used to confirm the expression of myc-tagged S fragment. (**D**) Minimal binding sequence for 7B2 to bind SARS-CoV-2 S protein was aligned with indicated beta-CoVs. The potential essential residues for mAb binding based on their cross-reactivity were denoted in a box. (**E**) Site-directed mutagenesis was performed to generate alanine substitution in myc-tagged S fragment of SARS-CoV-2. 293FT cells were transfected with alanine mutants and subjected to Western blot analysis. Anti-myc antibody was used to confirm the expression of myc-tagged wild-type and mutated S fragments.

To identify the binding sites for 7B2, 20-mer peptides with 15 overlapping amino acids from the extended HR2 region of SARS-CoV-2 WT S protein were synthesized and the binding of mAbs to peptides was determined by ELISA. The sequences of these peptides are shown in Supplementary Table S2. 7B2 bound significantly to peptide WH8-19 and WH8-20, containing residues 1138–1157 and residues 1143–1162. In contrast to 7B2, S2P6 bound peptide WH8-19, WH8-20 and WH8-21 (Figure 3A). Unlike S2P6, 7B2 did not bind to peptide WH8-21; thus, its epitope is distinct from S2P6. Alignment of WH8-19 and WH8-20 suggested that motif P_1143_ELDSFKEELDKYFK_1157_ are the minimal binding sequence for 7B2, whereas alignment of WH8-19, WH8-20 and WH8-21 indicated that residues 1148–1157 are essential for the binding of S2P6 (Figure 3B). This finding is in agreement with a previous study by Pinto et al. in 2021, which demonstrated that S2P6 bound the motif F_1148_KEELDKYF_1156_ [18].

The abilities of mAbs 7B2 and S2P6 to bind different beta coronaviruses were then compared. 293FT cells were transfected with myc-tagged S fragments of human coronavirus (HCoV)-OC43, MERS-CoV, bat coronavirus (BtCoV)-HKU5 and mouse hepatitis virus (MHV), followed by Western blot analysis (Figure 3C). In accordance with previous studies, S2P6 cross-reacted with S fragments of OC43, MERS-CoV, HKU5 and MHV. On the other hand, 7B2 only cross-reacted with OC43 and MHV. Thus, the cross-reactivity of 7B2 is different from S2P6 due to its ability to make contacts with different residues in S.

Multiple sequence alignment of minimal binding sequence of 7B2 with other beta-CoVs indicated that residues K1149, E1150 and D1153 in SARS-CoV-2 WT S might be crucial for 7B2 binding (Figure 3D). Thus, site-directed mutagenesis was performed to substitute the potential residues to alanine and WB was performed to check the binding of mAbs to the mutated S fragments (Figure 3E). Single substitution of K1149A or D1153A diminished the binding of 7B2 to S fragment, indicating that these two residues in S are essential for interacting with 7B2. In contrast, K1149A did not affect the binding of S2P6, while D1153A substitution slightly weakened the binding of S2P6. E1150A substitution did not affect the binding of either 7B2 or S2P6.

### 3.4. Comparison of Mode of Inhibition by mAbs 7B2 and S2P6

**Figure 4:**
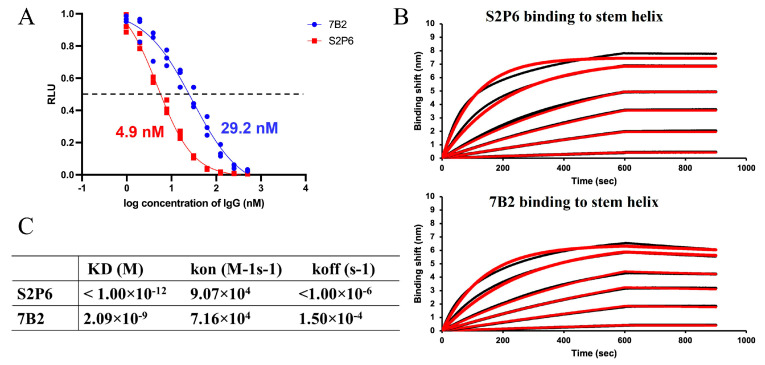
Inhibition on membrane fusion and binding with stem helix of 7B2 and S2P6. (**A**) 7B2 and S2P6 were tested in cell–cell fusion assay, displaying inhibitory activity with IC50 of 29.2 nM and 4.9 nM, respectively. (**B**) Binding of 7B2 and S2P6 upon stem helix was evaluated using BLI assay. Sensorgrams were acquired with the antibody at varied concentrations, 1, 2, 10, 20, 50 and 100 nM. Experimental data is displayed in black, with fitting curves displayed in red. (**C**) Dissociation constant K_D_, association rate kon and dissociation rate koff quantified based on the BLI assay are summarized in the table.

To further evaluate the mode of inhibition of mAb 7B2, a cell–cell fusion assay was carried out to quantify the effects of 7B2 and S2P6 on the fusogenic activity of the SARS-CoV-2 S protein. Upon the S-mediated membrane fusion, alpha and omega fragments were combined to form a functional beta-galactosidase, which produced a luminescence signal after reaction with substrate to indicate fusogenic activity. A dose-dependent decrease in the activity was observed in the presence of 7B2 and S2P6 (Figure 4A), suggesting that both antibodies are able to block S-mediated membrane fusion specifically. Consistent with observation in MNT and PRNT assays, 7B2 displayed an IC50 of 29.2 nM, weaker than S2P6 with an IC50 of 4.9 nM.

To further investigate the interaction, the binding of 7B2 and S2P6 with a peptide of stem helix (1141–1161), which is designed based on SARS-CoV-2 WT S, was analyzed through BLI assay. As the stem helix is highly conserved, the residues 1141–1161 in the S gene of B.1 virus (used in cell–cell fusion assay) and EG.5.1.1 virus (used in MNT and PRNT) are identical to that of SARS-CoV-2 WT. Binding kinetics were quantified based on responses upon 7B2 or S2P6 binding to the stem helix immobilized on the sensor chip (Figure 4B). The association rate of 7B2 was similar to S2P6, while the dissociation of 7B2 was faster than S2P6 by more than 100-fold. The dissociation constant (K_D_) of 7B2 was 2.09 nM (Figure 4C). Given the flat dissociation curve of S2P6, K_D_ could not be quantified accurately, estimated to be lower than 1 pM (Figure 4C). The weaker binding affinity of 7B2 was consistent with the milder inhibitory activity and neutralization potency.

### 3.5. AlphaFold3 Prediction of the Structure of 7B2 Binding to Stem Helix

**Figure 5:**
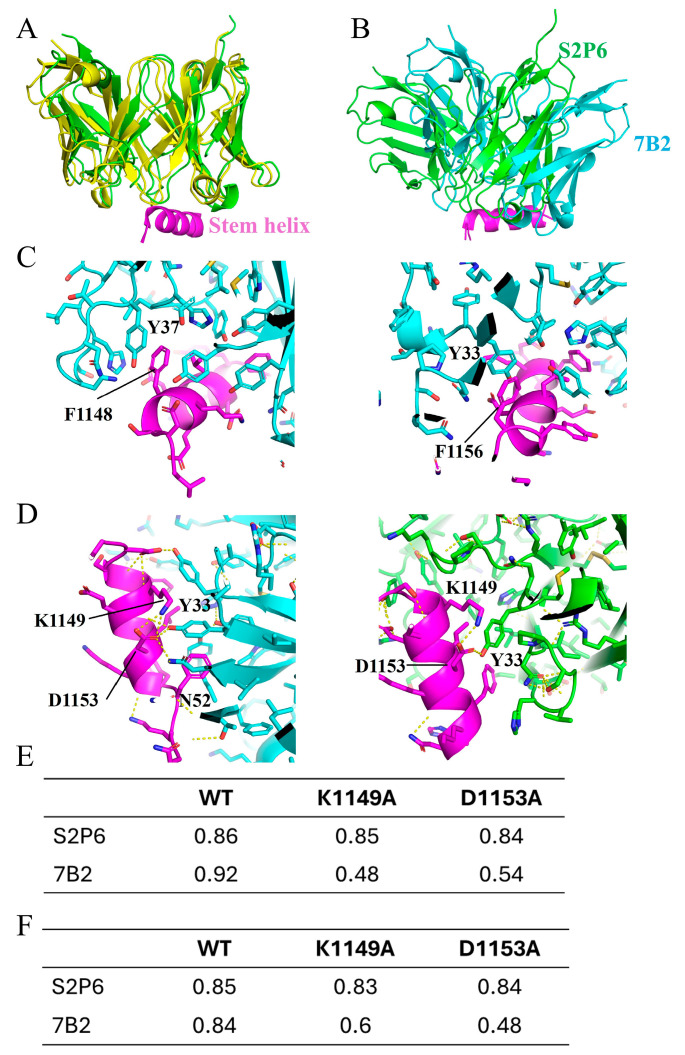
Structures of 7B2 and S2P6 binding to stem helix. (**A**) Comparison between AlphaFold3 (AF3)-generated structure (green) and X-ray crystal structure (yellow, PDB ID: 7rnj) of S2P6 binding to stem helix. Structures are displayed in cartoon representation. Stem helix is colored in purple. (**B**) AF3-generated structure of 7B2–stem helix complex is aligned based on the stem helix, superimposed to AF3-generated structure of S2P6-stem helix complex. 7B2 antibody is displayed in light blue. S2P6 antibody is displayed in green. Stem helix is displayed in purple. (**C**) Interface between 7B2 and stem helix. Side chains are displayed in stick representation. Critical π–π interactions between stem helix F1148 and light chain Y37 (left), and stem helix F1156 and heavy chain Y33 (**right**) are indicated as labeled. (**D**) K1149 and D1153 of stem helix contribute to binding with 7B2 by forming network of polar interactions with Y33 and N52 of 7B2 heavy chain (**left**). K1149 and D1153 of stem helix are less involved in polar interaction when binding with S2P6, with only one interaction between D1153 and Y33 of S2P6 heavy chain (right). In (**A**–**D**), only structure generated with no glycosylation at N1158 are displayed to avoid repetition. (**E**) Confidence score, ipTM, generated by AF3 for structures of 7B2 and S2P6 binding with wild-type stem helix and mutant K1149A and D1153A. (**F**) Confidence score, ipTM, generated by AF3 for structures of 7B2 and S2P6 binding with glycosylated wild-type stem helix and mutant K1149A and D1153A. N-acetylglucosamine was added to N1158 of stem helix as input for AF3.

To study the antibody–antigen interface in detail, structures of stem helix-bound 7B2 and S2P6 were generated by AlphaFold3 (AF3) [23]. Sequences of scFv for 7B2 and S2P6 were uploaded together with the stem helix sequence (with and without glycosylation at N1158) for AF3 prediction. The reliability of the prediction was evaluated based on template modeling scores, namely pTM and ipTM. For all the predicted structures, the pTM scores were higher than 0.85, suggesting a high reliability for the overall structure. The ipTM score indicates the quality of prediction on protein complex, measuring the accuracy of the interaction between the scFv and stem helix. The ipTM scores were 0.92 and 0.86 for 7B2 and S2P6, respectively. For the glycosylated stem helix, predicted structures for both 7B2 and S2P6 showed no significant difference from those without glycosylation, with ipTM 0.84 and 0.85, respectively (Supplementary Figure S1). To further validate the AF3-predicted structure, the predicted structure of S2P6 was compared with the experimental structure (PDB ID: 7rnj) of stem helix-bound S2P6 Fab determined by X-ray crystallography (Figure 5A). Superimposing the two structures showed that the predicted structure largely resembled the X-ray structure of S2P6. Collectively, the AF3 predictions are highly reliable, closely representing the native structure.

The structure of the 7B2–stem helix complex suggested that 7B2 binds to a similar epitope on the stem helix but with an angle different from S2P6 (Figure 5B). A similar set of residues on the stem helix were involved in 7B2 binding. Hydrophobic residues, F1148, L1152 and F1156, are buried toward the binding interface. Notably, there was a large number of aromatic rings located at the antibody interface, contributing significantly to interactions with stem helix. Y37 of light chain and Y33 of heavy chain interact with F1148 and F1156, respectively, forming the π–π interactions at the interface (Figure 5C). Compared with S2P6, 7B2 accesses stem helix from an orientation tilted by around 30 degrees. As a result, K1149 and D1153 interact with 7B2, forming a network of polar interactions with Y33 and N52 of 7B2 heavy chain (Figure 5D). Upon S2P6 binding, K1149 and D1153 contribute much less involvement. Mutations, K1149A and D1153A, were found able to disrupt 7B2 binding. Structures of 7B2 binding with K1149A and D1153A were predicted with extremely low ipTM scores in AF3 prediction for both glycosylated and non-glycosylated stem helixes (Figure 5E,F), suggesting an unreliable interaction caused by disrupted binding interface. On the contrary, S2P6 binding was not affected by the two mutations, as indicated by the high ipTM scores (Figure 5E,F). The predicted impact of these mutations is in good agreement with the mutagenesis studies (Figure 3E), demonstrating the accuracy of the AF3-generated structures.

## 4. Discussion

The persistent emergence of new SARS-CoV-2 variants with greater transmissibility and immune evasion remains a global public health threat. The substantial number of mutations in the S protein, especially RBD, limits the effectiveness of antiviral agents or vaccines that target RBD-ACE2 interaction. The first generation of approved SARS-CoV-2 vaccines was developed based on the S gene of the ancestral Wuhan-Hu-1 strain and they have been found to be less effective against emerging VOCs, particular Omicron, due to multiple mutations that reduce the binding of anti-S neutralizing antibodies [27,28,29]. To overcome this, the two widely used messenger RNA (mRNA)-based vaccines from Pfizer/BioNTech and Moderna have to be updated with the S sequences of newly emerged variants [30,31,32]. The other alternatives, like inactivated virus or recombinant protein-based vaccines, also did not yield neutralizing antibodies of sufficient breadth to effectively protect against Omicron and its variants. For example, inactivated virus vaccines such as BBIBP-CorV, CoronaVac and BBV152 are the early approved vaccines, with BBIBP-CorV showing 78.1% efficacy and CoronaVac showing 50.7% efficacy for preventing symptomatic COVID-19 in phase III trials. However, the neutralization activities of sera from individuals vaccinated with Sinopharm or Sinovac are limited towards Omicron [29,33,34]. NVX-CoV2373 is a recombinant nanoparticle vaccine composed of the full-length spike glycoprotein from the prototype SARS-CoV-2 strain. It demonstrated an efficacy of 89.7% against the Alpha (B.1.1.7) variant [35] but the neutralization activity of NVX-CoV2373 antibody is significantly reduced against the Omicron variant in vaccinated individuals [36].

Unlike the RBD, the S2 subunit is markedly more conserved and could be an ideal target for the development of antiviral agents that combat newly emerging variants. In the present study, we generated mAb 7B2 by immunizing mice with an immunogenic region comprised HR2 domain as well as the linker between HR1 and HR2. 7B2 exhibited neutralizing activity against Omicron subvariant EG.5.1.1. Epitope mapping revealed that 7B2 bound the P_1143_ELDSFKEELDKYFK_1157_ motif, which is located in the stem helix (SH) region in S2 subunit. SH is located upstream of HR2, with a high degree of conservation across diverse beta-CoVs. In the prefusion SARS-CoV-2 S protein, SH is made up of a 20-residue-long α-helix and the SH of three S protomers form a helical bundle at the membrane-proximal domain. In the S2 postfusion structure, residues at the N- and C-terminal of SH uncoil, while the middle part remains folded as a short surface-exposed helix [37]. A number of SH-targeting, neutralizing mAbs, including S2P6, have been isolated or elicited from natural infection and vaccination, as well as murine immunization with a combination of S antigens from different beta-CoVs. The binding of mAbs to SH disrupts the prefusion S confirmation and inhibits the formation of a six-helix bundle (6-HB) which is essential for membrane fusion. Despite their similar inhibitory mechanism, these neutralizing mAbs bind SH with distinct binding modes and orientations. They also exhibit varying cross-reactivity and cross-neutralization [38].

By peptide ELISA and site-directed mutagenesis, mAb 7B2 was shown to bind to SH and its mode of inhibition was compared to another published mAb S2P6 which was derived from a COVID-19 convalescent patient [18]. It can cross-react and neutralize diverse beta-CoVs including SARS-CoV, MERS-CoV and HCoV-OC43, as well as multiple SARS-CoV-2 variants. S2P6 targets motif F_1148_KEELDKYF_1156_, and F_1148_, E_1151_, L_1152_, D_1153_, Y_1155_ and F_1156_ are the essential residues for its binding. Our results show that mAb 7B2 binds to S in a distinct manner from S2P6 because the alanine substitution of K1149A and D1153A abolishes the interaction of S with 7B2 but has no impact for S2P6. Consistently, when AlphaFold3 was used to compare the structures of stem helix bound 7B2 and S2P6, the results show that these two residues in S are important for interacting with 7B2 but not S2P6. The AF3 predictions are highly reliable and reveal that 7B2 binds to a similar region on the stem helix but at a different angle from S2P6.

Despite that, the-AF3 predicted structures may be biased by the training dataset, which has included X-ray structure of S2P6 complex. With this limitation, the AF3 prediction may only focus on the most stable conformation and inefficiently evaluate the contribution of protein dynamics on binding. Protein dynamics simulation analysis could be performed in the future to provide more comprehensive analysis on the antibody binding.

Although 7B2 has weaker binding affinity to S and lower virus neutralizing capacity than S2P6, it has a similar mode of inhibition by blocking membrane fusion. Our results show that the difference between 7B2 and S2P6 is due to their contact with distinctive residues in the SH of S2 domain. Previously, a structural comparison of another murine mAb named B6, which neutralizes MERS-CoV but not SARS-CoV-2 [39], with S2P6 revealed that they bound the same epitope with very distinct orientations, indicating that binding orientation is a crucial determinant for neutralization breadth [18]. Similarly, our AF3 prediction shows that 7B2 also binds to the same region as S2P6 but at a different orientation and makes contact with different residues in the SH which explains why 7B2 binds to SARS-CoV-2 but not MERS-CoV. Thus, these three mAbs (S2P6, B6 and 7B2) have different neutralizing activities against SARS-CoV-2 because they bind to the same helical region (F_1148_KEELDKYF_1156_) in the SH at different orientations. Indeed, this helical region has been described as a supersite of vulnerability because multiple broadly neutralizing anti-S mAbs have been identified to bind to it [40].

Numerous neutralizing S2 mAbs binding to different functional motifs have been isolated from COVID-19 patients or immunized mice [41,42], indicating that the S2 subunit contains multiple immunogenic sites and an S2-based vaccine candidate could potentially induce polyclonal antibodies binding to different functionally important and highly conserved motifs in S2 [38]. Although mAbs targeting S2 have lower neutralizing activities than those binding to the receptor binding domain in S1, an S2-based vaccine could block viral fusion effectively if the polyclonal antibodies act in combination or show synergism. Indeed, several studies have demonstrated that S2 targeting vaccine in different forms elicited broad neutralizing against SARS-CoV-2 VOCs in animal models. Ng et al. have revealed that immunization using a DNA vaccine encoding membrane-bound wild-type SARS-CoV-2 S2 subunit (residues 686–1211) protected mice against wild-type and alpha variant challenge [43]. Furthermore, immunization of mice using an mRNA vaccine encoding S2 subunit with deletion of glycosites induced cross-reactive immune response against both alpha- and beta-CoVs [43,44]. Pang et al. reported that vaccination using a subunit vaccine HR121-targeting HR1 domain achieved protection against prototype SARS-CoV-2 in various animal models including hACE2 transgenic mice, Syrian golden hamsters and rhesus macaques [44,45].

## 5. Conclusions

In this study, we have generated and characterized a novel murine neutralizing mAb 7B2 binding to the stem helix in the S2 subunit of SARS-CoV-2. Taken together with other published mAbs [18,39,40], different neutralizing mAbs are able to bind to this stem helix at different angles, which influences the residues in S making critical contact with each of the mAbs. Thus, the conservation of antibody epitopes as well as the angle of bound mAbs contribute to the breadth of these mAbs towards VOCs of SARS-CoV-2 as well as other beta coronaviruses. In-depth understanding of the mechanisms of action of a large variety of neutralizing mAbs targeting different functional motifs in the S protein will be important for the development of pan-beta coronaviruses vaccines that could also protect against pre-emergent zoonotic strains [41,46]. Recent studies have developed new strategies to increase the breadth of coronavirus vaccine protection and some of these incorporate different antigens or multiple immunogenic epitopes [27,28,29]. For example, Mosaic-8b, which contains RBD from SARS-CoV-2 and seven animal coronaviruses, is capable of inducing broader immune response in mice and non-primates including Delta variant and SARS-CoV-1 [47]. GBP511 also contains RBD from SARS-CoV-2 and different animal coronavirus, and showed protection against animal sarbecoviruses and SARS-CoV-1 infection [48]. MigVax-101 is a multi-antigen vaccine that contains RBD and two domains of viral nucleocapsid (N) from SARS-CoV-2 that is able to immunize BALB/c mice and Sprague Dawley rats with oral vaccination with three doses [49]. RBD-scNP is a nanoparticle vaccine composed with helicobactor pylori ferritin with 24 copies of RBD attached with sortase A [50]. It is a designed β-coronavirus/merbecovirus vaccine. It showed broad neutralizing antibodies capable of recognizing animal coronavirus, SARS-CoV-1, MERS-CoV and SARS-CoV-2 strains [27,50]. These finding suggest that vaccines designed with different variant-derived antigens have the potential to enhance cross-neutralization and broad-spectrum protection against SARS-CoV-2. Thus, further studies may also add different S2-based fragments into these new platforms to stimulate cross-reactive anti-S2 neutralizing antibodies and increase the breadth of protection.

## Data Availability

All the data supporting the findings of this study are available within the main manuscript and the Supplementary Materials. Further inquiries can be directed to the corresponding author.

## Supplementary Materials

The following supporting information can be downloaded at: https://www.mdpi.com/article/10.3390/vaccines13070688/s1, Table S1: Amino acid sequence comparison of S fragments in the S2 domain of spike proteins of SARS-CoV-2 and other beta coronaviruses; Table S2: Amino acid sequences of the synthetic peptides used in ELISA and BLI; Figure S1: Comparison of AF3 predictions performed with and without glycosylated stem helix.
