# Supplementary material for: Characterization of the Binding and Inhibition Mechanisms of a Novel Neutralizing Monoclonal Antibody Targeting the Stem Helix Region in the S2 Subunit of the Spike Protein of SARS-CoV-2
Source: Vaccines (Basel). 2025 Jun 26;13(7):688. doi: 10.3390/vaccines13070688 (PMC12299175; doi:10.3390/vaccines13070688)
Supplement: Supplementary file 1 [file vaccines-13-00688-s001.zip › vaccines-3677277-supplementary.pdf]

Supplementary Table S1. Amino acid sequence comparison of S fragments in the S2 domain of spike proteins of SARS-CoV-2 and other beta coronaviruses.

No.	Virus and accession number of S protein	VOC	Year of deposited sequence	% amino acid identity of S fragment[1]	
				Residues 686 to 1047	Residues 1048 to 1206
1	SARS-CoV-2_YP_009724390.1	Wild type	2019	100	100
2	OC43_YP_009555241.1	NA[2]	2001	44.13	36.31
3	MERS_YP_009047204.1	NA[2]	2012	46.81	33.12
4	HKU5_9EH8	NA[2]	2025	44.88	38.61
5	MHV_6VSJ	NA[2]	2020	44.41	28.3
6	B.1_LC769019	Wild type	2023	100	100
7	B.1.351_7LYQ	Beta	2021	99.72	100
8	B.1.1.7_7EDJ	Alpha	2021	98.9	99.37
9	P.1_QUA55751.1	Gamma	2021	99.72	99.37
10	B.1.525_QWM26293.1	Eta	2021	99.72	100
11	B.1.429_7N8H	Elipson	2021	98.34	100
12	B.1.621_WKQ70944.1	Mu	2021	99.72	100
13	C.37_8VYF	Lambda	2024	97.79	100
14	B.1.1.529_7QO9	Omicron	2021	97.79	100
15	BA.2_7XOD	Omicron	2022	97.24	100
16	BA.1_7XO5	Omicron	2022	97.79	100
17	BA.2.12.1_7XNS	Omicron	2022	96.96	100
18	BA.2.75_8GS6	Omicron	2022	96.41	100
19	BA.4_7XNQ	Omicron	2022	97.24	100
20	BA.5_WGR38744.1	Omicron	2022	98.9	100
21	BQ.1.1_8XI6	Omicron	2023	97.24	100
22	XBB.1_8IOU	Omicron	2023	97.24	100
23	XBB.1.5_WHJ03660.1	Omicron	2023	98.9	100
24	BA.2.12.1_8R8K	Omicron	2023	98.62	100
25	EG.5.1_8XMT	Omicron	2023	97.24	100
26	EG.5.1.1_EPI_ISL_19016298	Omicron	2023	98.62	100
27	JN.1_8Y5J	Omicron	2024	96.96	99.37
28	LP.8.1_EPI_ISL_19810969	Omicron	2025	98.62	98.1
29	LF.7.2.1_EPI_ISL_19704142	Omicron	2025	98.62	99.37
30	NP.1_EPI_ISL_19819986	Omicron	2025	98.62	98.73
31	NP.1.8.1_EPI_ISL_19864412	Omicron	2025	98.62	98.37

[1]% computed relative to SARS-CoV-2_YP_009724390.1.

[2]NA: "not applicable"

Supplementary Table S2. Amino acid sequences of the synthetic peptides used in ELISA and BLI. All of them were designed according to SARS-CoV-2 WT (accession number: YP_009724390.1).

Peptide	Sequence
WH8-1	HLMSFPQSAPHGVVFLHVTY
WH8-2	PQSAPHGVVFLHVTYVPAQE
WH8-3	HGVVFLHVTYVPAQEKNFTT
WH8-4	LHVTYVPAQEKNFTTAPAIC
WH8-5	VPAQEKNFTTAPAICHDGKA
WH8-6	KNFTTAPAICHDGKAHFPRE
WH8-7	APAICHDGKAHFPREGVFVS
WH8-8	HDGKAHFPREGVFVSNGTHW
WH8-9	HFPREGVFVSNGTHWFVTQR
WH8-10	GVFVSNGTHWFVTQRNFYEP
WH8-11	NGTHWFVTQRNFYEPQIITT
WH8-12	FVTQRNFYEPQIITTDNTFV
WH8-13	NFYEPQIITTDNTFVSGNCD
WH8-15	DNTFVSGNCDVVIGIVNNTV
WH8-16	SGNCDVVIGIVNNTVYDPLQ
WH8-17	VVIGIVNNTVYDPLQPELDS
WH8-18	VNNTVYDPLQPELDSFKEEL
WH8-19	YDPLQPELDSFKEELDKYFK
WH8-20	PELDSFKEELDKYFKNHTSP
WH8-21	FKEELDKYFKNHTSPDVDLG
WH8-22	DKYFKNHTSPDVDLGDISGI
WH8-23	NHTSPDVDLGDISGINASVV
WH8-24	DVDLGDISGINASVVNIQKE
WH8-25	DISGINASVVNIQKEIDRLN
WH8-26	NASVVNIQKEIDRLNEVAKN
WH8-27	NIQKEIDRLNEVAKNLNESL
WH8-28	IDRLNEVAKNLNESLIDLQE
WH8-29	EVAKNLNESLIDLQELGKY
SH peptide used in BLI	LQPELDSFKEELDKYFKNHTS

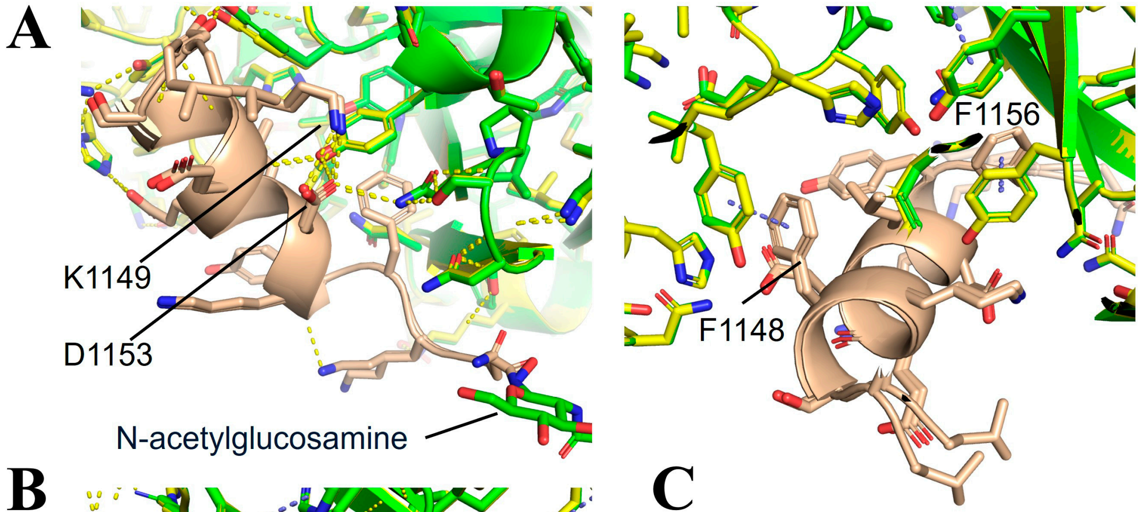

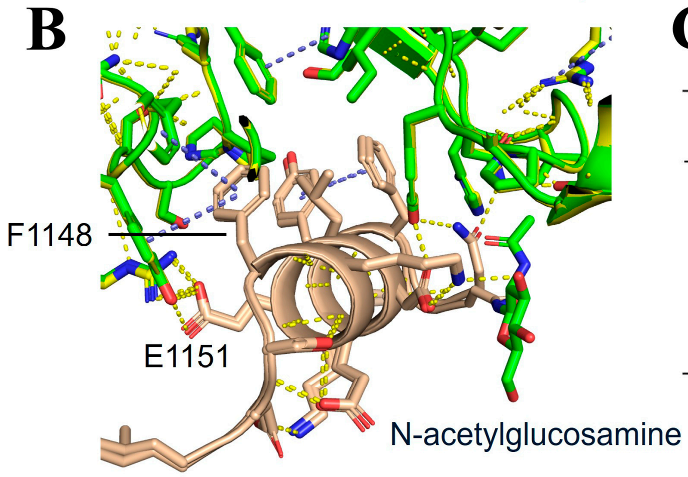

C

	S2P6	7B2
WT	0.85	0.84
K1149A	0.83	0.6
D1153A	0.84	0.48

Supplementary Figure S1. Comparison of AF3 predictions performed with and without glycosylated stem helix. (A) Comparison between AF3 predicted 7B2 bound structures of non-glycosylated (yellow) and glycosylated (green) stem helix. (B) Comparison between AF3 predicted S2P6 bound structures of non-glycosylated (yellow) and glycosylated (green) stem helix. In (A) and (B), polar interactions have been indicated by yellow dashed lines, and pi-pi interactions have been coloured in light blue. (C) Table of ipTM scores generated by AF3 for S2P6 and 7B2 binding with wild type (WT) and mutant stem helix
